# Neural mechanisms of audio tactile integration in the flutter range

**DOI:** 10.1186/1471-2202-12-S1-P62

**Published:** 2011-07-18

**Authors:** Mario Pannunzi, Alexandre Pereda Banos, Alexis Perez Bellido, Salvador Soto-Faraco, Gustavo Deco

**Affiliations:** 1 Department of Technology, Universitat Pompeu Fabra, Barcelona, Spain; 2 Barcelona Media, Barcelona, Spain; 3 Department of Psychology, Universitad de Barcelona, Barcelona, Spain

## 

**Figure 1:**
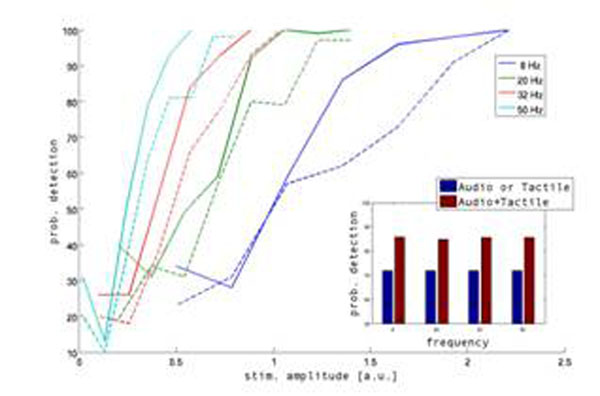
Model's detection probability for different stimulus amplitude and frequency (8,20,32,50 Hz). Dashed line is when is present only one stimulus (audio or tactile), solid line when the two senses are combined. Inset: Differences of detection probability when there is/are one or two stimulus combined (see results of [4]).

Unveiling the mechanisms that integrate sensory information from different modalities has become a main aim of psychological research in the last decade. In this project we are interested in the specific case of auditory-tactile interactions in the perception of vibrotactile events, an aspect that has not received as much attention as audio-visual or visuo-tactile interactions. The case of audio-tactile interactions deserves special attention. As both modalities respond to the same physical property (mechanical pressure), it’s tempting to predict that such coincidence would allow integrative interactions at a variety of levels of processing, including the earliest ones. In fact, there is ample evidence that tactile events can influence activity in the earliest stages of auditory processing and there are several behavioral demonstrations of auditory influences on tactile perception. Particularly, vibrations in the flutter range (~10-50 Hz) constitute an interesting case for study given that vibratory patterns within this range are represented in the firing rates of the neurons in the sensory cortices for both somatosensory and auditory stimuli. Moreover in this range we have well detailed information given by the neurophysiological experiments. In particular studies describing the neural correlates of perceptual detection of vibrotactile events on the flutter range (e.g. see [2]) show that neurons in S1 respond with a very high degree of fidelity to the temporal profile of the stimulus. Similar results have been achieved with audio stimuli (e.g. see [3]) always on the flutter range. Our intention is to compare human behavioral data with an appropriate computational model in order to offer an appropriate hypothesis on the processing stage or stages where this audio-tactile interactions take place. Thus, we want to test if integration between somatosensory and auditory information does indeed take place on the flutter range as it does for high frequencies [4]. To this aim, we implement the same experiment of [4] for the flutter range; the experiment consists on comparing the detection threshold for audio, tactile and the two senses at the same time. As we want to ascertain whether stimuli integration takes place before or after perceptual detection, we back the behavioral data with a computational analysis of the processes involved by means of a network model composed of integrate-and-fire neurons. The neural network consists of multiple interacting layers, with the hypothesis that the integration happens at a high level processing areas in accordance with recent neurophysiological results [5]. The model can be applied both to flutter and no-flutter ranges. Our preliminary results (see figure 1) are in accordance with the behavioral results of [4] (our experimental results are not conclusive).
